# Pelvic Tilt Angle Differences Between Symptom-Free Young Subjects and Elderly Patients Scheduled for THA: The Rationale for Tilt-Adjusted Acetabular Cup Implantation

**DOI:** 10.2174/1874325001812010364

**Published:** 2018-08-31

**Authors:** Carlos J. Marques, Tobias Martin, Andrzej Kochman, Adrian Goral, Frank Lampe, Viktor Breul, Josef Kozak

**Affiliations:** 1 Research Center of the Orthopaedic and Joint Replacement Department at the Schoen Klinik Hamburg Eilbek, Dehnhaide 120, D-22081 Hamburg, Germany; 2 Navigation Lab, Aesculap AG, Am Aesculap-Platz, D-78532 Tuttlingen, Germany; 3 Medical Scientific Affairs, Aesculap AG, Am Aesculap-Platz, D-78532 Tuttlingen, Germany; 4 Orhtopaedic and Joint Replacement Department at the Schoen Klinik Hamburg Eilbek, Dehnhaide 120, D-22081 Hamburg, Germany; 5 Faculty of Life Sciences at the Hamburg University of Applied Sciences, Lohbrügger Kirchstraße 65, D-21033 Hamburg, Germany; 6 AGH University of Science and Technology, Krakow, Poland; 7 Trauma and Orthopedic Department, Hospital of the Ministry of Internal Affairs, Wroclaw, Poland

**Keywords:** Ultrasonography, Computer assisted surgery, Total hip replacement, Anterior pelvic plane, Pelvic tilt, Hip arthroplasty (THA)

## Abstract

**Background:**

The question whether Pelvic Tilt (PT) angles measured in the supine position are adequate for the alignment of the acetabular cup without an adjustment for anatomical differences between patients is of clinical importance. The aim of this work was to test for factors that can significantly affect PT angles.

**Methods:**

In the present retrospective cohort comparison, the PT angles of 12 Symptom-Free Young Subjects (SFYS) and 45 patients scheduled for Total Hip Arthroplasty (THA) were compared. The data was collected during two studies with the use of a novel smartphone-based navigated ultrasound measurement system. Multi-factorial analysis of variance was run to determine which factors significantly affect PT.

**Results:**

Body position (F= 126.65; P< 0.001) and group (SFYS vs. THA patients) (F= 17.52; P< 0.001) had significant main effects on PT. There was also a significant interaction between body position and group (F= 25.59; P< 0.001). The mean PT increased by 8.1° from an interiorly to a neutral tilted position (P< 0.001) and 21.4° from a neutral to a posteriorly tilted position (P< 0.001) with the transition from the supine into the upright position for the SFYS and THA patients, respectively.

**Conclusion:**

In both groups, PT changed significantly with a transition from the supine to the upright position. A position-dependent mean PT increase in the patient group showed that acetabular cup alignment based on PT in the supine position is not reliable without taking into consideration the inclination of the pelvis in standing position. This may lead to instability and dislocations.

## 1 INTRODUCTION

Hip dislocation is a major cause of complications after Total Hip Arthroplasty (THA), with reported prevalence rates ranging from 0.3 to 3% [1]. According to the annual report of the Swedish hip register, dislocation was the second most common reason for revision, responsible for 25% of all reoperations within the first year after THA [2].

Acetabular component alignment is considered to play a determinant role in THA dislocation [3]. According to Lewinnek *et al*. the acetabular component should be placed within a 40° ± 10° inclination and a 15° ± 10° anteversion safety zone, inside which the greatest range of motion of the hip with the minimum dislocation risk should be achieved [4].

**Fig. (1):**
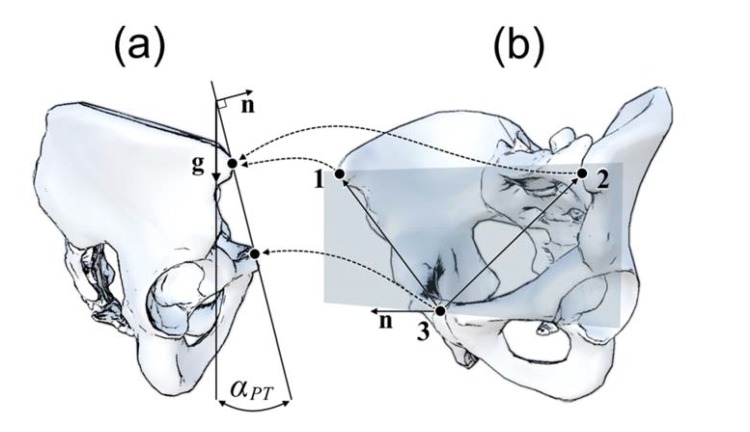
(a) Pelvic tilt angle (αPT), defined as the angle between the APP and the gravity vector in upright position, and (b) Anterior Pelvic Plane (APP), defined as the plane through the right and left anterior superior iliac spines (1 and 2) and the symphysis pubis (3).

The Anterior Pelvic Plane (APP) is defined as the plane through the right and left anterior superior iliac spines (R-ASIS and L-ASIS) and the Symphysis Pubis (SP) (Fig. **1b**). Traditionally, the APP has been used as a reference plane for implantation of the acetabular component during non-navigated THA. The angle between the APP and the coronal (frontal) plane is defined as pelvic tilt angle (PT) (Fig. **1a**). The PT angle can assume negative (anterior PT) and positive (posterior PT) values and it provides information about the spatial orientation of the pelvis.

Mayr and colleagues measured the APP of 120 patients in supine and upright position and calculated their PT angles. They found a mean PT angle of 5.6° in supine and 6.7° in upright position. The authors concluded that the APP in supine position is a valid reference for acetabular cup implantation, since in their study, mean PT angles were almost equal in both positions [5]. In contrast, recent studies have shown significant PT differences between measurements performed in supine and upright position [6-9]. Therefore, the question whether PT angles measured in the supine position are adequate for acetabular cup alignment without an adjustment or without considering individual patient differences is highly relevant [10]. The accurate knowledge of the spatial orientation of the pelvis in an upright position is extremely important during acetabular cup implantation (mostly performed in supine position), because the pelvis position influences the final hip cup inclination and anteversion angles [6, 11, 12]. The inclination and anteversion angles have been advocated to influence the outcomes and survival rates in THA [13]. In the past, malposition of the acetabular cup was related to higher dislocation rates, limb length discrepancy, femoroacetabular impingement, aseptic loosening and earlier revisions [3, 4, 14, 15].

In a study by Au *et al*. the pelvis of 30 THA patients was tilted more posteriorly in upright than supine position thus leading to a significant increase in acetabular cup anteversion and inclination (p< 0.001). Furthermore, the orientation of the acetabular cup was significantly more likely to be found outside the Lewinnek safe zone [4] in upright than in supine position (p< 0.001) [6].

The functional orientation of the pelvis in different body positions also depends on the flexibility of the lumbar spine. Buckland *et al*. investigated the effect of lumbar spine fusion on the dislocation rates after THA and found that patients that previously underwent lumbar spinal fusion had increased rates of dislocation after THA [16].

In view of the above-referred findings the data of two previous studies [17, 18] were treated in a secondary data analysis. The aim was to test for factors that significantly affect PT angles. Gender, physician, trial, body position, group affiliation (symptom free young subjects vs. hip arthritis patients scheduled for THA), age and BMI were used as independent factors.

## 2 MATERIALS AND METHODS

### 2.1 Study Design

In this secondary data analysis a retrospective cohort comparison design was used. The data used for the present purpose was collected during two studies with the use of the same measurement system. One study investigated the intra- and inter-rater reliability of a navigated ultrasound system in the assessment of PT in Symptom-Free Young Subjects (SFYS) [18]. The second study assessed PT in patients scheduled for THA [17]. The data of the study with the SFYS was analyzed and published previously [19] and was now compared with the data of the patients scheduled for THA. In each publication a individual research question was investigated.

The Medical Ethics Commission of the Federal State of Hamburg, Germany, approved the research proposal with the SFYS (File PV5216) and the local Bioethical Committee of the Wroclaw Medical University, Wroclaw, Poland, approved the research proposal for the study with the patients (File RNN/191/15/KE).

### 2.2 Subjects

The study sample consists of 45 (23 female and 22 male) patients scheduled for THA and a convenience sample of 12 SFYS (8 women and 4 men).

Before participating all subjects were required to read and sign an informed consent form. For the SFYS the inclusion criteria were age (the subjects should be between 18 and 30 years old) and symptoms (the subject should have no hip or lower limb pathologies in their medical record, and should be free from acute health problems). The inclusion criteria in the study with the patients were: unilateral or bilateral osteoarthritis of the hip with a radiologic score 3 or 4 on the Kellgren-Lawrence scale. Patients with ankylosing spondylitis were excluded from the study.

### 2.3 Measurement System

**Fig. (2):**
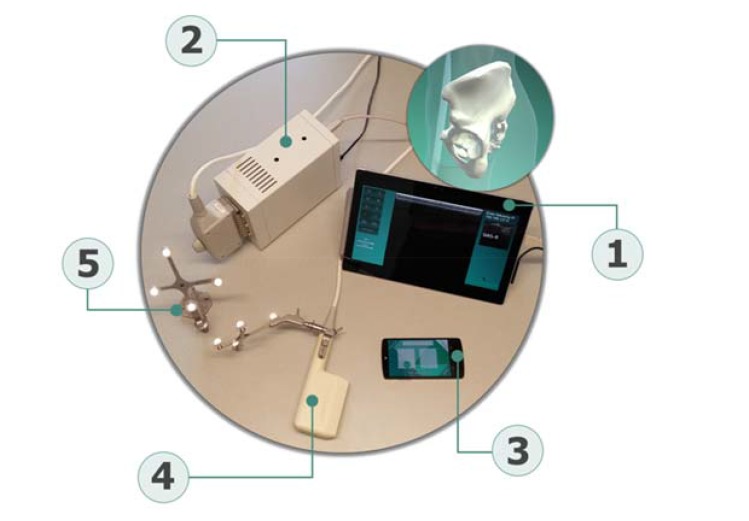
Components of the measurement system: (1) central unit (Microsoft Surface, Redmond, USA), (2) Ultrasound device (Echo Blaster 128, Telemed, Vilnius, Lithuania), (3) handheld tracking system (iPhone 6), (4) ultrasound transducer with attached rigid body, (5) reference rigid body (Aesculap AG, Tuttlingen, Germany).

The measurement system used for PT measurements in both studies is composed of a newly developed tracking software application that runs on a commercial smartphone (iPhone 6, Apple Inc., Cupertino, USA), referred further on as smart-localizer unit, two sets of reflective markers called “rigid bodies” for position tracking (Aesculap AG, Tuttlingen, Germany), a certified ultrasound device (Echo Blaster 128, Telemed, Vilnius, Lithuania) attached to a trackable 3-7 MHz linear ultrasound transducer with a sound window of 80 mm, and a commercial tablet (Microsoft Surface, Redmond, USA) as central unit. The tablet runs a custom-built software that enables communication with the other components and performs the necessary calculations (Fig. **2**). The intra-rater reliability of the system when measuring PT angles was good to excellent and moderate to excellent for the supine and upright positions, respectively. Inter-rater reliability remained below expected values, probably due to the imaging protocols, which were probably still not described with enough detail [18]. This system was chosen because it is noninvasive and offers a high degree of flexibility, since the tracking unit is held by hand, allowing the measurement of PT in different positions.

**Fig. (3):**
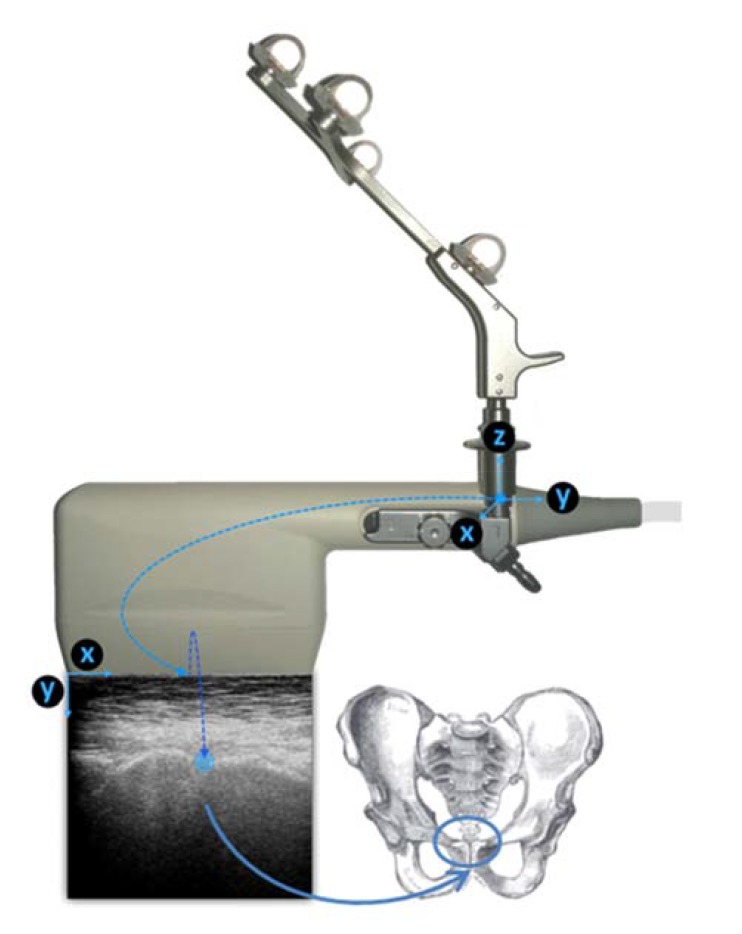
Ultrasound transducer with attached rigid body, enabling to receive the actual 3D position and orientation of the transducer, with the ultrasound picture of the symphysis pubis.

The alignment of the APP is computed based on the bony landmarks digitized with the use of the ultrasound transducer and the smart localizer unit. The ultrasound transducer is attached to a rigid body that allows the localizer to determine its position and orientation (Fig. **3**). During the measurements, the operator records ultrasound images of the three bony landmarks that are necessary to define the APP (R-ASIS, L-ASIS, SP). Once the acquisition is complete, the operator has to identify the right position of the landmarks on each image. Using the position of the landmark in each image and the position of the probe during its acquisition (recorded by the localizer) the system computes the 3-dimensional coordinates of the landmarks and consequently the spatial position of the APP. The positions of the landmarks are computed in a reference coordinate system in association with a reference rigid body that remains stationary during the measurement. In the final step, the orientation of the APP is determined with respect to the gravity vector, obtained from the built-in inertial sensors of the smart localizer. The detailed description of the system and the algorithms used is presented in a previous publication [20].

**Fig. (4):**
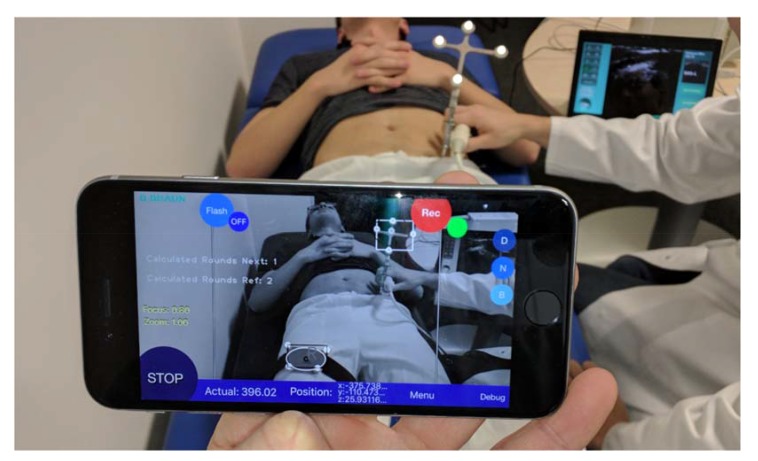
Tracking unit, ultrasound transducer with attached rigid body and reference rigid body while tracking the L-ASIS in the supine position.

The tracking unit does not need to be attached to a fixed tripod and can be held by hand, which offers a high degree of flexibility (Fig. **4**). Due to this, the position of the tracking environment can be changed arbitrarily and unfavorable measuring positions can be easily circumvented.

### 2.4 Procedures

The SFYS who agreed to participate were scheduled for PT assessments. Two physicians carried out the measurements independently. The PT of each subject was measured three times consecutively in supine and upright position. A total of three PT measurements per subject and per physician were available for the present data analysis.

The PT of the patients scheduled for THA was measured in the same way, firstly in the supine position and then in an upright position.

### 2.5 Statistical Analysis

Mean ± Standard Deviation (SD) values were used to characterize the groups.

A multi-factorial analysis of variance model (MANOVA) was run to test for factors which significantly affect PT (dependent variable). The following independent factors were considered in the model: Physician (Physician 1, Physician 2 and Physician 3), Trial (Trial 1, Trial 2 and Trial 3), Body Position (Supine, Upright), Gender (Female, Male), Group (Group 1: SFYS, Group 2: Patients), Age and BMI. A backward elimination method was used with an elimination limit of 0.1. For all tests, the level 0.05 was accepted as the criterion for statistical significance.

All statistical tests were carried out with the SAS software version 9.4 (SAS Institute Inc., Cary, NC, USA).

## 3 RESULTS

The demographic data of the subjects included in the study is shown in Table **1**. The subjects in the patient group were significantly older (P< 0.001) and had a significantly higher BMI (P< 0.001) in comparison with the SFYS.

The final model was reached in three steps. Body position (F= 126.65; P< 0.001) and group affiliation (F= 17.52; P< 0.001) had significant main effects on PT. There was also a significant interaction between body position and group (F= 25.59; P< 0.001).

**Fig. (5):**
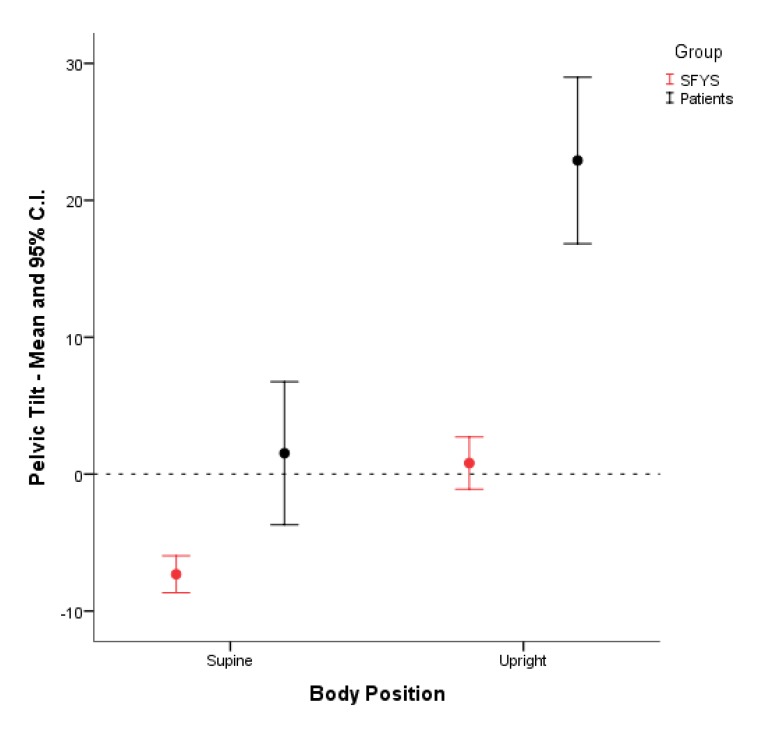
Mean (95% C.I.) pelvic tilt values of the subjects in both groups (SFYS vs. patients) by body position. Negative PT value = anterior tilt, positive PT value = posterior tilt.

In both groups the transition from the supine into the upright position was associated with a significant mean PT increase. The mean PT increased by 8.1° (P< 0.001) and 21.4° (P< 0.001) with the transition from the supine into the upright position for the SFYS and patient, respectively. In supine position the mean PT of the SFYS was tilted anteriorly (-7.3° ± 5.7). In contrast, the mean PT of the patients was tilted posteriorly in the supine position (1.5° ± 17.3). In upright position the mean PT of the SFYS was nearly in neutral position (0.8° ± 8.1), while the pelvis of the patients was strongly posteriorly tilted (22.9° ± 20.2) (Fig. **5**).

## 4 DISCUSSION

In most cases THA is performed with the patient in supine position. While in non-navigated THA surgeons normally use the APP and other anatomical structures as reference for acetabular component alignment, some computer navigation systems rely on the PT angle, which is also calculated based on the APP. The question whether PT measured solely in supine position is adequate for acetabular cup alignment was investigated in the present study. The main findings of the present study are the following: PT angles of SFYS were significantly different from the ones of patients scheduled for THA, in both, the supine and upright position. Furthermore, within each group the pelvic tilt changed significantly with a transition from the supine into the upright position. The pelvis of the subjects scheduled for THA was tilted posteriorly in supine position. Their mean posterior tilt increased by 21.4° (P< 0.001) with the transition from the supine into the upright position. This means that the acetabular cup placed within the safe zone in supine position is probably outside the safe zone in upright position.

Independently from the body position, there are also patients-specific PT differences, which are reflected in the large standard deviations showed in Table **1**. This suggests that individualized acetabular cup alignment may be necessary to improve stability and decrease dislocation rates in THA.

Acetabular cup anteversion is affected by the pelvic orientation, particularly by the PT angle. While an anteriorly tilted pelvis increases, a posteriorly tilted pelvis decreases the anteversion angle of the cup [8]. At the same time, the hip range of motion and the range of motion of the lumbar spine influence the degree of pelvic tilt too [9, 21].

Recently, Seagrave *et al*. presented a systematic review of studies that assessed the risk of dislocation after primary THA [22]. Most of the included studies did not identify differences between dislocating and non-dislocating THA in regard to the mean cup anteversion and inclination angles. Furthermore, no significant reductions in dislocation rates were achieved in the studies where the acetabular cup was aligned within the Lewinnek’s safe zone. Another study concluded that the “historical target values” for acetabular cup inclination and anteversion may be useful but should not be considered a safe zone, since the majority of the THAs dislocated within those target values [23]. In view of these findings the following question arises: why is the safety zone defined by Lewinnek not safe? The data presented in the present study provides a possible explanation: an acetabular cup aligned within the safe zone in supine position is not necessarily inside the safe zone in upright or in sitting position. The body position influenced PT significantly in both, young symptom free subjects and in patients scheduled for THA. Since the anteversion angle of the acetabular cup is significantly affected by PT, changes in body position may lead to instability and dislocation.

PT values of age-matched controls without hip arthritis were not available for the present retrospective data analysis. It would be interesting to test whether the PT values of patients scheduled for THA would differ from the ones of the age matched controls. This is considered by the authors as a study limitation and should be investigated in a further study. Also it would be of interest to investigate how PT changes over time before and after THA. The measurement system used is suitable for such purposes since it is noninvasive and not associated with x-ray radiation exposition.

The present results provide a possible explanation for the high percentage of THA dislocations in patients, whose acetabular component was found to be within the safety zone. In view of the present results, PT-based acetabular cup alignment performed in supine position needs to be adjusted individually. The adjustment should consider the individual amount of PT-change, which can be measured by a transition from the supine into the upright position.

## CONCLUSION

The PT of symptom free young subjects is significantly different from the PT of patients scheduled for primary THA. In both groups the PT changed significantly with a transition from the supine into the upright position. A position-dependent mean PT increase of 21.4° (P< 0.001) in the patient group showed that acetabular cup alignment based on PT measured in supine position is not reliable without an individual adjustment. Hip cup alignment based on PT values acquired solely in supine position may lead to instability and dislocation in other body positions.

## Figures and Tables

**Table 1:** Demographic and pelvic tilt (PT) data.

	SFYS	Patients	Mean Diff. (P-Value) [95% C.I.]
Number of patients (n)	12 (8F; 4M)	45 (23F; 22M)	
Age (years)	24.2 ± 3.2	67.0 ± 10.7	42.8 (P< 0.001) [-45.6 to 40.0]
BMI (Kg/m^2^)	23.0 ± 1.3	28.7 ± 4.4	5.6 (P < 0.001) [-6.7 to -4.6]
PT Supine position (deg)	-7.3 ± 5.7	1.5 ± 17.3	8.8 (P= 0.02) [-13.3 to -4.5]
PT Upright position (deg)	0.8 ± 8.1	22.9 ± 20.2	22.1 (P< 0.001) [-28.4 to -15.7]

Values are mean ± SD. SFYS= Symptom free young subjects; F= Female; M= Male

## LIST OF ABBREVIATIONS

PT: Pelvic Tilt
SFYS: Symptom Free Young Subjects
THA: Total Hip Arthroplasty
APP: Anterior Pelvic Plane
R-ASIS: Right Anterior Superior Iliac Spine
L-ASIS: Left Snterior Superior Iliac Spine
SP: Symphysis Pubis
